# Three-dimensional approaches to measuring primary cilia in hippocampal neurons: A comparative analysis

**DOI:** 10.1093/jnen/nlag001

**Published:** 2026-01-27

**Authors:** Sofia Rasmusson, Seyedeh Marziyeh Jabbari Shiadeh, Nour Dada, Ellen Åkesson, Carina Mallard, Maryam Ardalan

**Affiliations:** Department of Physiology, Institute of Neuroscience and Physiology, Sahlgrenska Academy, University of Gothenburg, Gothenburg, Sweden; Department of Physiology, Institute of Neuroscience and Physiology, Sahlgrenska Academy, University of Gothenburg, Gothenburg, Sweden; Department of Physiology, Institute of Neuroscience and Physiology, Sahlgrenska Academy, University of Gothenburg, Gothenburg, Sweden; Department of Physiology, Institute of Neuroscience and Physiology, Sahlgrenska Academy, University of Gothenburg, Gothenburg, Sweden; Department of Physiology, Institute of Neuroscience and Physiology, Sahlgrenska Academy, University of Gothenburg, Gothenburg, Sweden; Department of Physiology, Institute of Neuroscience and Physiology, Sahlgrenska Academy, University of Gothenburg, Gothenburg, Sweden; Department of Clinical Medicine, Translational Neuropsychiatry Unit, Aarhus University, Aarhus, Denmark

**Keywords:** hippocampus, IMARIS, neuron, primary cilia, stereology

## Abstract

Primary cilia are non-motile sensory organelles that detect extracellular signals; disruptions in their functions are linked to neurodevelopmental disorders. Because cilia lengths can rapidly change in response to stressors, they are important for both plasticity and brain homeostasis. Accurate measurement of ciliary length is therefore essential but the absence of standardized methods and the variability introduced by different techniques can compromise measurement reliability and precision. To address this challenge, our study employed two distinct methods to estimate the length of primary cilia in hippocampal subregions in mice. We compared stereology-based 3D quantification, which is considered a methodological gold standard due to its unbiased sampling design and correction for tissue shrinkage, with 3D reconstruction to measure primary cilia length. 3D reconstruction imaging used a 100× oil-immersion objective. With neuronal cilia typically ∼2-10 µm long in hippocampus, a 1-µm z-step provided multiple optical sections per cilium thereby ensuring full structural visualization. Our findings show that both methods allow simple and equally precise measurements of neuronal primary cilia length in hippocampal subregions. Their strong agreement provides researchers with reliable tools for studying primary cilia on immunohistochemically stained sections and supports a consistent methodological framework for investigating cilia dynamics.

## INTRODUCTION

Neurons require effective ways of registering and communicating changes in the environment for adaptive behavior. Primary cilia are packed with receptors that respond to extracellular signals and modify downstream signaling events.^1–3^ In the brain, disruption in ciliary signaling pathways has repeatedly been linked to altered plasticity and behavioral abnormalities.^4–7^ Primary cilia vary widely in length depending on cell type and brain region, typically ranging from a few micrometers to over 10 µm in some neuronal and glial populations.^6^^,^^8–10^ Additionally, the lengths of the primary cilia change in response to physiological stressors, including inflammatory, hormonal and metabolic signals.^10–13^ Cilia dynamics are strongly influenced by intraflagellar transport (IFT), which supports cilium assembly and maintenance by mediating the bidirectional movement of protein cargo along axonemal microtubules.^14^ However, IFT is not the only mechanism that determines cilia length. A growing body of evidence shows that cilia length is shaped by multiple interacting factors, including the rate of the transport of structural and signaling components along the cilium by the IFT machinery, axonemal tubulin turnover and stability,^15^ membrane trafficking and ciliary membrane composition,^16^ and intracellular signaling pathways that modulate axoneme growth and resorption.^17^^,^^18^ Together, these processes contribute to the dynamic regulation of cilia lengths beyond IFT alone.

Nonetheless, appropriate modification of cilia length seems to contribute to both plasticity and maintaining homeostasis in the brain, ie, disrupted primary cilia formation and function have been associated with neurodevelopmental disorders, collectively known as ciliopathies.^19^^,^^20^ Proper measurement of primary cilia length is therefore essential for understanding the impact of these trafficking events on ciliary function and related cellular mechanisms.

Neuronal primary cilia were first identified more than four decades ago through electron microscopy studies of mammalian brain tissue.^21^^,^^22^ Although this method laid the foundation for future studies on cilia, it was limited in its application to routine analyses. The identification of cilia-specific proteins such as type III adenylyl cyclase (ACIII), improved the field by providing reliable immunolabeling markers for neuronal primary cilia.^23^ ACIII is currently the most extensively used marker for studying neuronal primary cilia in rodents. Primary cilia can be identified with immunofluorescence labeling in vitro and in vivo enabling direct measurement in two-dimensional (2D) images. In brain regions with densely packed neurons or overexposed background staining, however, whole ciliary length can be difficult to measure using 2D images in which accuracy and precision can be compromised due to bias and errors in individual intra- and inter-variability.^24^^,^^25^ Notably, differences in GPCR localization across neuronal nuclei are associated with regional variability in cilia composition and signaling; some of these pathways may indirectly influence cilia length.^8^^,^^24^^,^^26^ These differences indicate the complexity and variability of primary cilia making accurate length measurement challenging and pointing to a critical need for optimized methods to address these disparities and enhance our understanding of cilia functions. Thus, accurately measuring cilia length is technically challenging due to issues with reproducibility and user error. Artificial intelligence (AI) offers promising solutions by reducing inconsistencies in image analysis, such as cilia length measurement, co-localization, and fluorescence intensity.^24^ However, training AI to accurately recognize specific patterns such as those of primary cilia requires significant time and user expertise. Moreover, AI training demands consistent image quality in terms of background and cilia visibility to ensure accurate results. To address these challenges, we conducted a dual-method comparison for estimating neuronal primary cilia length in mouse hippocampal subregions. We employed two distinct and advanced methods to achieve this, ie, 3D Quantification (Stereology) and 3D reconstruction.

3D quantification stereology is widely used as the methodological gold standard for morphometric analysis because it provides unbiased and accurate estimates within the processed tissue. Stereology accounts for tissue shrinkage by measuring the final section thickness and positioning the optical disector height entirely within the true z-dimension, eliminating systematic underestimation caused by shrinkage and less sensitive to the type of staining (immunohistochemical [IHC] versus immunofluorescence staining).^27^ The 3D reconstruction approach is used to is primarily used in in vitro studies in which cell numbers are lower and cell density is much less. It is especially common in studies involving immunofluorescence-stained sections. Application of this method of image stacking, segmentation on IHC-stained sections allows visualization of the entire primary cilia thereby accounting for its true spatial orientation. This method also employs z-stack imaging and filament tracing to reconstruct primary cilia in 3D space but does not correct for tissue shrinkage.

By comparing these two approaches, our goal was not to determine absolute biological accuracy since true in vivo cilium length after fixation is unknowable but rather to evaluate methodological accuracy, precision, and agreement. This dual-method approach not only enhances our understanding of the structural characteristics of neuronal primary cilia but also provides insights into the effectiveness of different measurement tools. The ultimate goal of the study was to establish a more robust and reproducible methodology for neuronal primary cilia length measurement on IHC-stained sections, which could be critical for future research on cilia function and its implications in neuronal and cellular processes.

## METHODS

### Animals

Experiments on these mice were approved by the Animal Ethics Committee of Gothenburg University (Dnr: 2487/19) and were conducted in the animal facilities of Experimental Biomedicine, University of Gothenburg. C57BI/6J wild type mice were purchased from Janvier Labs (Le Genest-Saint-Isle, France), bred and housed with a 12-h light/dark cycle in room temperature (20-22°C). All methods were performed and reported in accordance to the ARRIVE guidelines.^28^ Mice had ad libitum access to food (standard laboratory chow diet by B&K, Solna, Sweden) and drinking water. Brain tissues from six male mice at the age of postnatal day (PND 45 ± 5) were analyzed.

### Brian tissue collection and IHC

The mice were deeply anesthetized via intraperitoneal administration of pentobarbital (Pentacour, 150 mg/kg^−1^). Right or left hemisphere of brain was selected randomly and immersed in a 6% buffered formaldehyde (Histofix; Histolab products AB, Västra Frölunda, Sweden) at 4°C for 2 weeks. In the next step, hemispheres were put in a 48-h cryoprotective solution (30% sucrose, w/v^−1^) before freezing using isopentane (Sigma Aldrich) and then cut into 40‐μm‐thick coronal sections, with a cryostat (Leica, CM 3050 S, Germany). Sections including hippocampus were selected by a systematic sampling principle and section-sampling fraction of 1/8,^29^^,^^30^ for the neuronal primary cilia staining. Free-floating 40-µm-thick sections were washed in phosphatase-buffered saline (PBS), then incubated at 85°C target retrieval solution (Dako, Glostrup, Denmark) for 40 min, followed by washing with PBS for 2 × 10 min. For blockage, sections were put in endogenous peroxidase [3% H_2_O_2_ in PBS, Sigma Aldrich for 10 min, then washed in PBS with 0.25% Triton-X-100 (PBS-T), Sigma Aldrich], before incubation in polyclonal primary rabbit anti-ACIII (EnCor Biotechnology Inc, Cat# RPCA-ACIII, 1:10.000) at 4°C overnight. In the second day, brain sections were incubated in room temperature with polyclonal secondary biotinylated goat-anti-rabbit (1:250, Vector Laboratories, Olean, NY, USA) and PBS-T for 2 h. Then, sections were washed with PBS-T, before incubation in the ABC Elite solution was prepared by mixing 1.5% of reagent A (avidin-biotinylated enzyme complex) and 1.5% of reagent B (biotinylated horseradish peroxidase) in PBS, according to the manufacturer’s instructions (Vector Laboratories). After 1-h incubation in ABC elite solution, the sections were washed in PBS-T (2 × 10 min) and immunolabeled with 3,3-diaminobenzidine solution (Acros Organics, Geel, Belgium). Finally, the sections were washed in distilled water followed by counterstaining with thionin solution 0.25% (Sigma T3387) and dehydrated in a graded alcohol series of 95% and 99% ethanol, then cleared with xylene before coverslipping.

### Measurement of the neuronal primary cilia length

Both stereology-based 3D quantification and 3D reconstruction were performed on the same DAB-stained 40 µm section for each animal. Because the two methods analyzed identical sections, this approach eliminates anatomical or staining variability and allows direct comparison of cilia measured by each technique.

### 3D quantification (Stereology)

ACIII-thionin counterstained sections were used for 3D quantification of primary cilia length on pyramidal neurons in the CA1 and CA3 pyramidal cell layers (CA1P, CA3P) and on granular cells in granular cell layer (GCL) of both ventral and dorsal hippocampus. Analysis was performed using 100× oil-immersed lens on a light microscope modified for stereology with a digital camera (Leica DFC 295, Germany) and newCAST software (Visopharm, Hørsholm, Denmark). The actual post-processing section thickness was measured for each series. The optical disector height of 10 µm was placed entirely within this measured thickness, ensuring that all length estimates were corrected for z-axis shrinkage due to fixation and dehydration. This results in an unbiased estimate of primary cilia length that reflects the true dimensions of the processed tissue. Also, it allows live, continuous z-axis stereological measurements on the cilia at different lengths.

**Figure 1.**
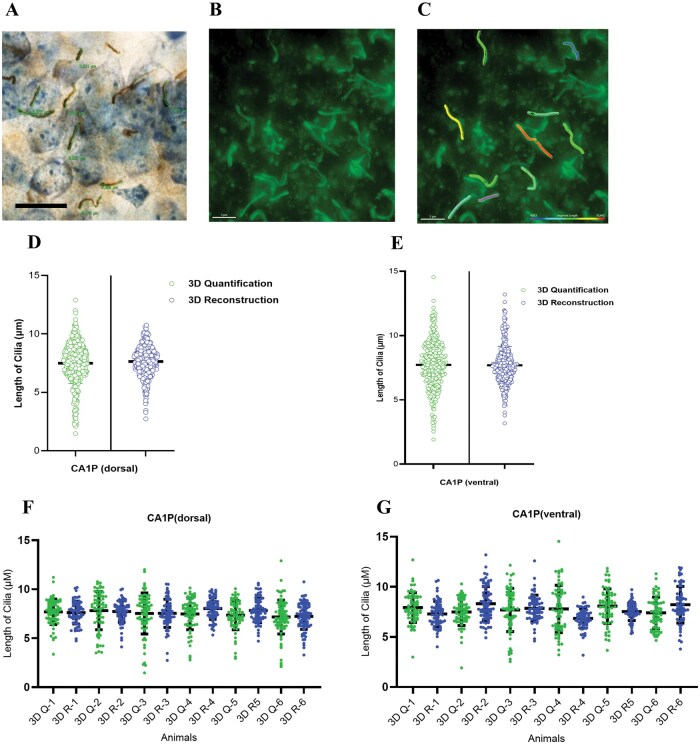
Comparison between 3D reconstruction and stereology-based 3D quantification of neuronal primary cilia length in the CA1 pyramidal layer (CA1P) hippocampal subregion of mice. (A) Example of 3D quantified primary cilia on DAB immunostained section using optical disector probe in NewCAST software using a 100× objective lens with ultimate resolution from light microscopy, Scale bar = 10 µm. (B) Example of ACIII-positive primary cilia from a DAB-stained section imported into Imaris software and visualized using the green intensity channel, Scale bar = 5 µm. (C) 3D reconstructed primary cilia using Filament Tracer algorithm in Imaris. Blue color indicates the shortest cilia length, and red color is an indicator of the longest cilia, Scale bar = 5 µm. (D) No difference in the measured length of primary cilia using two different methods in the CA1P.dorsal. Each point represents the length of a single primary cilium, and all measured cilia from all animals in each group are shown. (E) No difference in the measured length of primary cilia using two different methods in the CA1P.ventral. Each point represents the length of a single primary cilium, and all measured cilia from all animals in each group are shown. (F) Distribution of primary cilia length measured in the CA1P.dorsal hippocampus, shown at the individual-animal level. Each column represents one animal, and each dot represents a single primary cilium measured using either stereology-based 3D quantification (green) or 3D reconstruction (blue). Black horizontal bars indicate the mean cilia length per animal. (G) Distribution of primary cilia length measured in the CA1P.ventral hippocampus, displayed at the individual-animal level. Each column represents one animal, and each dot represents a single primary cilium measured using stereology-based 3D quantification (green) or 3D reconstruction (blue). Black horizontal bars indicate the mean cilia length per animal. Statistical test was paired *t*-test. *N* = 6. Each data set represents measurements from ∼70 primary cilia per region per method for each animal. Black bars represent the mean ± SD.

**Figure 2.**
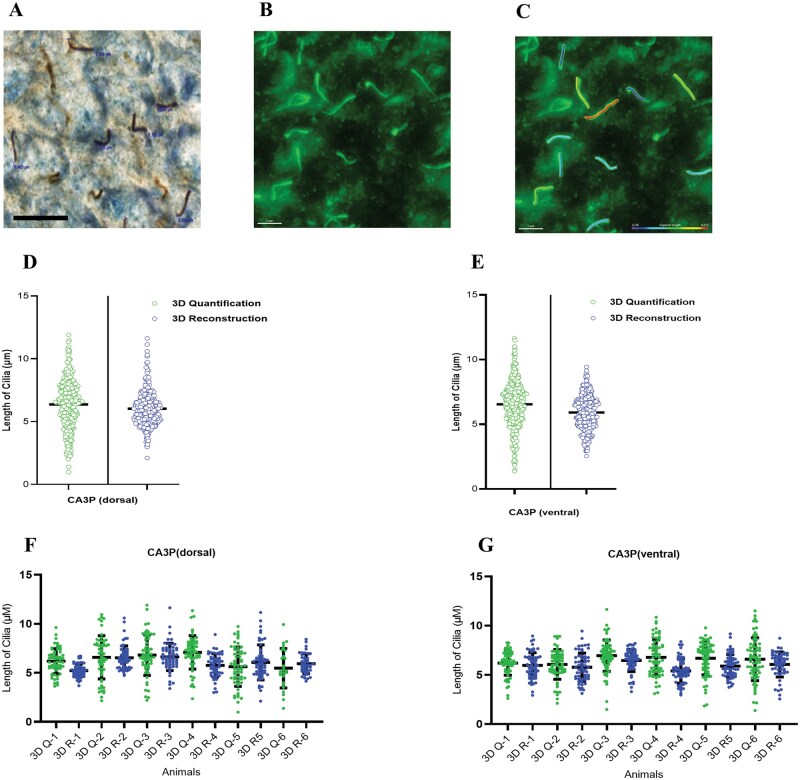
Comparison between 3D reconstruction and stereology-based 3D quantification of neuronal primary cilia length in the CA3 pyramidal layer (CA3P) hippocampal subregion of mice. (A) Example of 3D quantified primary cilia on DAB immunostained section using optical disector probe in NewCAST software using a 100× objective lens with ultimate resolution from light microscopy, Scale bar = 10 µm. (B) Example of ACIII-positive primary cilia from a DAB-stained section imported into Imaris software and visualized using the green intensity channel, Scale bar = 5 µm. (C) 3D reconstructed primary cilia using Filament Tracer algorithm in Imaris. Blue color indicates the shortest cilia length, and red color is an indicator of the longest cilia, Scale bar = 5 µm. (D) No difference in the measured length of primary cilia using two different methods in the CA3P.dorsal. Each point represents the length of a single primary cilium, and all measured cilia from all animals in each group are shown. (E) No difference in the measured length of primary cilia using two different methods in the CA3P.ventral. Each point represents the length of a single primary cilium, and all measured cilia from all animals in each group are shown. (F) Distribution of primary cilia length measured in the CA3P.dorsal hippocampus, shown at the individual-animal level. Each column represents one animal, and each dot represents a single primary cilium measured using either stereology-based 3D quantification (green) or 3D reconstruction (blue). Black horizontal bars indicate the mean cilia length per animal. (G) Distribution of primary cilia length measured in the CA3P.ventral hippocampus, displayed at the individual-animal level. Each column represents one animal, and each dot represents a single primary cilium measured using stereology-based 3D quantification (green) or 3D reconstruction (blue). Black horizontal bars indicate the mean cilia length per animal. Statistical test was paired *t*-test. *N* = 6. Each data set represents measurements from ∼60 primary cilia per region per method for each animal. Black bars represent the mean ± SD.

**Figure 3.**
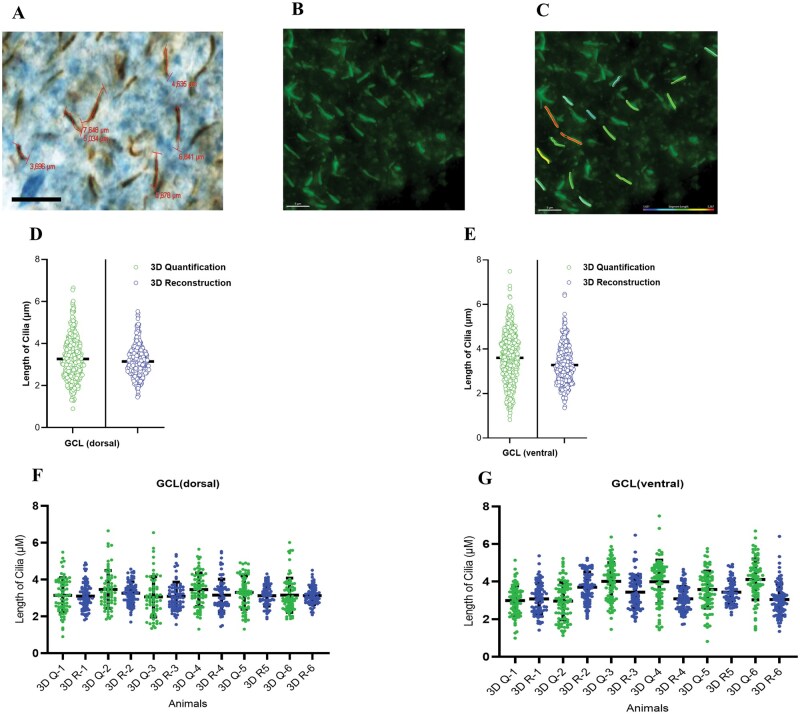
Comparison between 3D reconstruction and stereology-based 3D quantification of neuronal primary cilia length in the granular cell layer (GCL) hippocampal subregion of mice. (A) Example of 3D quantified primary cilia on DAB immunostained section using optical disector probe in NewCAST software using a 100× objective lens with ultimate resolution from light microscopy, Scale bar = 10 µm. (B) Example of ACIII-positive primary cilia from a DAB-stained section imported into Imaris software and visualized using the green intensity channel, Scale bar = 5 µm. (C) 3D reconstructed primary cilia using Filament Tracer algorithm in Imaris. Blue color indicates the shortest cilia length, and red color is an indicator of the longest cilia, Scale bar = 5 µm. (D) No difference in the measured length of primary cilia using two different methods in the GCL.dorsal. Each point represents the length of a single primary cilium, and all measured cilia from all animals in each group are shown. (E) No difference in the measured length of primary cilia using two different methods in the GCL.ventral. Each point represents the length of a single primary cilium, and all measured cilia from all animals in each group are shown. (F) Distribution of primary cilia length measured in the GCL.dorsal hippocampus, shown at the individual-animal level. Each column represents one animal, and each dot represents a single primary cilium measured using either stereology-based 3D quantification (green) or 3D reconstruction (blue). Black horizontal bars indicate the mean cilia length per animal. (G) Distribution of primary cilia length measured in the GCL.ventral hippocampus, displayed at the individual-animal level. Each column represents one animal, and each dot represents a single primary cilium measured using stereology-based 3D quantification (green) or 3D reconstruction (blue). Black horizontal bars indicate the mean cilia length per animal. Statistical test was paired *t*-test. *N* = 6. Each data set represents measurements from ∼70 primary cilia per region for each animal. Black bars represent the mean ± SD.

The region of interest was sampled with the area sampling frame (ASF) of 4.56%. We measured the lengths of primary cilia on randomly sampled 50-80 cilia per region per animal (Figures 1A, 2A, and 3A). Three-D quantification approach ensures that variations in cilia orientation and positioning are accounted for thereby providing a comprehensive view of their structural characteristics.

### 3D reconstruction

For 3D reconstruction of neuronal primary cilia, a systematic series of z-stacks of images was acquired with a z-plane step size of 1-μm (considering neuronal primary cilia length on average ranging from ∼2 to 10 µm in the analyzed hippocampal regions), with the middle section selected as zero. This was done on ACIII- IHC (DAB)-stained sections using a 100× oil-immersion lens (numerical aperture [NA] = 1.3) on a light microscope adapted for stereology (Figure S1).

The DAB reaction product was visualized and subsequently imported into IMARIS, in which the software assigns intensity values to a user-selected color channel (in this case, green) to enhance contrast for segmentation. Thus, the green appearance in reconstructed images reflects IMARIS pseudo-coloring rather than fluorescence imaging.

The selection criteria were that whole cilia length must be visible and distinguishable from other overlapping cilia or background staining. This acquisition procedure ensured capturing more than 3 primary cilia per image. The images were then analyzed using the Filament Tracers algorithm in the Imaris software (version 9.7; Bitplane AG, Zurich, Switzerland).

This method of analysis enabled the reconstruction of primary cilia from 3D captured images, providing an advantage for examining the detailed morphology and spatial relationships of primary cilia in their native context. Here, ACIII-stained IHC images were processed in the 3D reconstruction software to get a clear visualization of primary cilia in CA1P (Figure 1B and C), CA3P (Figure 2B and C) and GCL (Figure 3B and C). To assess intra-rater variability in 3D reconstruction of primary cilia, the same researcher independently measured the primary cilia three times using identical image sets.

### Statistical analysis

Statistical analyses were performed using SPSS (IBM Corp. Version 28.0. Armonk, NY, USA). Graphs were created in Prism 10 (GraphPad Software Inc., USA) and Sigmaplot 12.5 (SYSTAT, San Jose, CA) (Bland-Altman and difference [confidence interval]). Distribution of data was checked by making a Q-Q plot of the data before performing analysis. The variance homogeneity of data was also examined by Levene’s test. Statistical test for comparing two methods of quantification was paired *t*-test. Intra-variability in 3D reconstruction of primary cilia was tested by comparing three independent measurements using repeated measures analysis of variance (ANOVA). To assess the accuracy and reliability of the two methods for measuring primary cilia length, the analysis was performed independently by two researchers and data has been analyzed using paired-*t* test.

For agreement analysis, the individual cilia were identified within the same stereological sampling frames, and each cilium was measured using both stereology-based quantification (live z-stack focusing) and IMARIS 3D reconstruction (using a 3D image of the identical sampling frame). This allowed paired measurements suitable for Bland-Altman analysis. The data are presented as mean ± SD. A significance level of *P* ≤ .05 was applied in all analyses.

## RESULTS

In this study, we aimed to estimate the length of neuronal primary cilia within the dorsal and ventral CA1P, CA3P and GCL subregions of the hippocampus, regions characterized by a dense layer of pyramidal or granular cells. The pyramidal cells in the CA1P are notably smaller than those in the CA3P subregion but larger than the granular cells in GCL, which makes primary cilia more difficult to visualize in the 2D plane. We therefore approached length measurements of primary cilia using 3D quantification of IHC-sections as a gold standard and compared the results of this measurement with a 3D reconstruction method which allowed us to visualize the entire primary cilia, to account for their true spatial orientation and to provide insights into whether the two methods agreed with respect to precision and accuracy.

We measured the length of primary cilia in the ventral and dorsal hippocampus separately because these regions have distinct functional roles and may exhibit different cellular and molecular characteristics. The dorsal hippocampus is primarily associated with cognitive functions such as memory and spatial navigation whereas the ventral hippocampus is more involved in emotional regulation and stress responses. Given these functional differences, the structure and behavior of primary cilia may vary between the two regions, reflecting their specialized roles.

To achieve precise measurements and validate our approach, we utilized a dual-method strategy comparing stereology-based 3D quantification (NewCAST) and IMARIS software for 3D reconstruction. The measurements showed no significant differences in the estimated lengths of the neuronal primary cilia at both dorsal and ventral in CA1P (Figure 1D and E), in CA3P (Figure 2D and E) and GCL (Figure 3D and E). Comparing the methods on individual measurements of neuronal primary cilia length in ventral and dorsal hippocampus for each mouse showed no significant difference across all subregions (Figures 1F, G, 2F, G, 3F, and G). Thus, the two approaches exhibit strong precision and agreement. It is important to indicate that the 2D screenshots in Figures 1C, 2C, and 3C represent only one viewing angle; when the reconstruction is rotated, cilia that appear short in the xy view often reveal longer 3D trajectories. Therefore, orientation does not bias length estimation by applying 3D reconstruction of primary cilia.

To further visualize differences between methods at the individual-animal level, we include a Supplementary Figure in which each data point represents one animal, with color indicating measurement method (Figure S2). This representation demonstrates that primary cilia length estimates obtained by stereology-based 3D quantification and 3D reconstruction are highly comparable within each hippocampal subregion at both dorsal and ventral levels. Across CA1P, CA3P, and GCL, mean cilia lengths showed close overlap between methods, with no systematic deviation attributable to the measurement approach.

**Figure 4.**
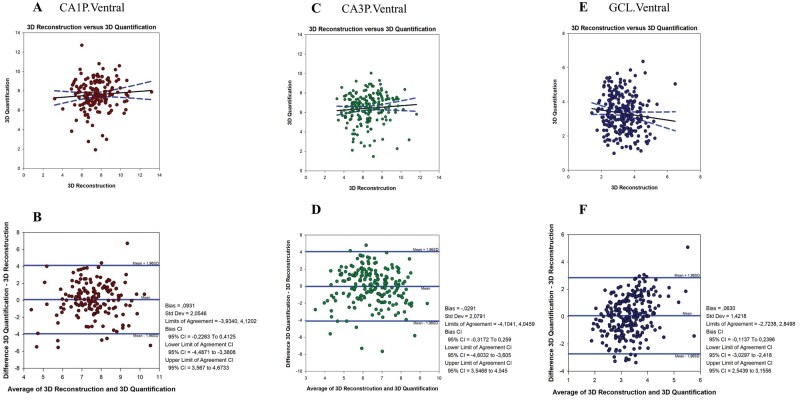
Bland-Altman analysis revealed a high degree of agreement between the two methods used for estimating primary cilia length at the ventral level of the hippocampus. Top: Method-comparison scatter plots showing the length of individual primary cilia measured using IMARIS 3D reconstruction (*X*-axis, µm) and stereology-based 3D quantification (*Y*-axis, µm). Each dot represents one cilium. (A, C, E). Bottom: Bland-Altman plot for data from two methods of primary cilia length measurements, including the limits of agreement. Blue lines represent mean bias and ±1.96 SD limits of agreement. *N* = 6 (B, D, F).

**Figure 5.**
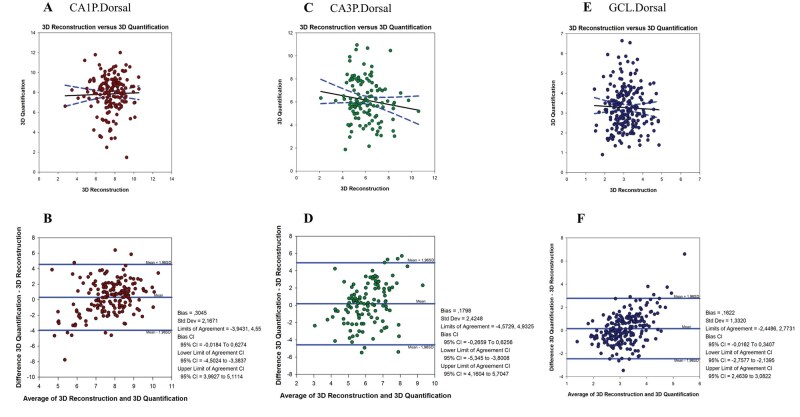
Bland-Altman analysis revealed a high degree of agreement between the two methods used for estimating primary cilia length at dorsal level of hippocampus. Top: Method-comparison scatter plots showing the length of individual primary cilia measured using IMARIS 3D reconstruction (*X*-axis, µm) and stereology-based 3D quantification (*Y*-axis, µm). Each dot represents one cilium (A, C, E). Bottom: Bland-Altman plot for data from two methods of primary cilia length measurements, including the limits of agreement. Blue lines represent mean bias and ±1.96 SD limits of agreement. *N* = 6 (B, D, F).

To evaluate the agreement between the two methods used for estimating primary cilia length in the CA1P, CA3P and GCL subregions of the hippocampus at both dorsal and ventral levels, we generated Bland-Altman plots. Because stereology corrects for shrinkage and 3D image reconstruction does not, the high level of agreement suggests that shrinkage did not differentially affect the interpretability of the two methods in assessing primary cilia length within the dense layer of neuronal cells in ventral (Figure 4A-C) and dorsal (Figure 5A-C) hippocampus. Most importantly, direct comparison with stereology the methodological gold standard involving live focusing through the full z axis showed no significant differences between methods.

Primary cilia length varied systematically across the hippocampal subregions at both dorsal and ventral levels. As summarized in Table S1, cilia were longest in the CA1 pyramidal layer, intermediate in CA3, and shortest in the granular cell layer (GCL). Importantly, these region-dependent patterns were consistent across both stereology-based 3D quantification and 3D reconstruction methods. Across methods, mean cilia length in CA1P ranged from approximately 7.5 to 7.7 µm, in CA3P from ∼5.9 to 6.5 µm, and in GCL from ∼3.2 to 3.6 µm. Minimum and maximum values, (as well as SDs), were highly comparable between methods within each subregion, indicating no systematic differences in estimated cilia length attributable to the measurement approach and reflecting intrinsic, region-specific structural characteristics of neuronal primary cilia. We also acknowledge that finer z-sampling may be required for other imaging modalities (eg, confocal fluorescence) or for structures approaching the diffraction limit.

The Bland-Altman plot depicted the differences in primary cilia length measurements obtained by NewCAST (3D quantification) and IMARIS software (3D reconstruction) against the average of these two methods. Our results indicated a consistent agreement between the two methods. The mean difference between the measurements was close to zero, indicating no systematic bias. Most of the differences fell within the limits of agreement (mean ± 1.96 SDs), suggesting that the discrepancies were within an acceptable range. The calculated limits of agreement indicated the range within which most of the differences between the methods were expected to lie (Figures 4D-F and 5D-F). The narrow limits further confirmed the reliability of the two methods in measuring primary cilia length. The limits of agreement observed in the Bland-Altman analyses reflect population-level variability rather than typical measurement error for individual cilia.

**Figure 6.**
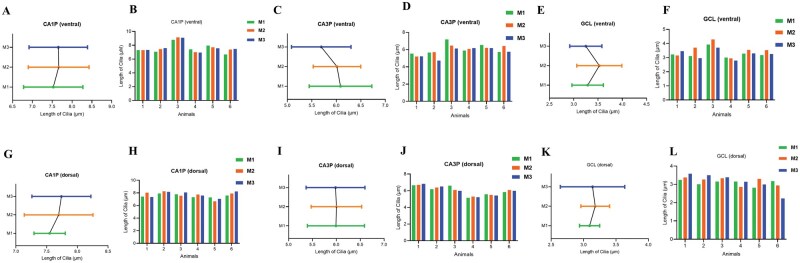
M1, M2, and M3 represent three independent 3D reconstructions of the cilia from the same image stacks, performed by the same researcher to assess intra-observer repeatability. (A) Total range of primary cilia length per measurement in the ventral CA1P subregion of the hippocampus. (B) Grouped bar plots illustrating the three individual measurements of primary cilia for each animal in ventral CA1P. (C) Total range of primary cilia length per measurement in the ventral CA3P. (D) Grouped bar plots illustrating the three individual measurements of primary cilia for each animal in ventral CA3P. (E) Total range of primary cilia length per measurement in the ventral GCL. (F) Grouped bar plots illustrating the three individual measurements of primary cilia for each animal in ventral GCL. (G) Total range of primary cilia length per measurement in the dorsal CA1P subregion of the hippocampus. (H) Grouped bar plots illustrating the three individual measurements of primary cilia for each animal in dorsal CA1P. (I) Total range of primate cilia length per measurement in the dorsal CA3P. (J) Grouped bar plots illustrating the three individual measurements of primary cilia for each animal in dorsal CA3P. (K) Total range of primary cilia length per measurement in the dorsal GCL. (L) Grouped bar plots illustrating the three individual measurements of primary cilia for each animal in dorsal GCL. Statistical test was repeated measure ANOVA. *N* = 6. The data are presented as mean ± SD.

Additionally, we tested repeatability and reproducibility of the 3D reconstruction method between three measurements by the same researcher and found no significant difference in primary cilia length across the three measurements in all hippocampal subregions. In the CA1P at ventral level, measurements (M)2 and M3 exhibit slightly longer cilia compared to M1, which is not significant (Figure 6A). The individual animal data, indicating consistency in the cilia length distribution across all six animals, with no significant outliers (Figure 6B). In the CA3P at ventral level, measurement of the primary cilia length, showing a non-significant decrease in cilia length for M3 compared to M2 and M1 (Figure 6C), but overall uniformity within each group (Figure 6D). Regarding ventral GCL, M2 exhibits non-significant longer cilia compared to M1 and M3 (Figure 6E), which was in the same pattern for individual mouse (Figure 6F). At the dorsal level of hippocampus, slight increase in cilia length is observed in M2 compared to M1 and M3 at CA1P with consistency across all animals within each group (Figure 6G and H). In the CA3P dorsal, relatively consistent cilia length across animals in all three groups was observed (Figure 6I and J) and in the dorsal GCL, the measurements showed longer cilia in M3, followed by M2 and M1 which was not significant (Figure 6K) and individual animal data highlighted consistent results within each group (Figure 6L). When we summarize the results of three measurements in ventral and dorsal hippocampus, we could see that in the ventral CA1P and GCL regions, M3 consistently exhibited longer cilia compared to M1 and M2. Conversely, the dorsal CA3P region shows less variation in cilia length among the groups. This consistency in measurements suggest minimal intra-individual variability in primary cilia length.

**Figure 7.**
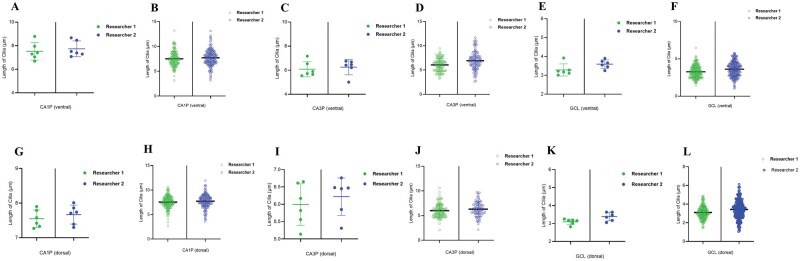
Assessment of inter-observer reliability in measuring primary cilia length across hippocampal subregions. Primary cilia length was quantified independently by two researchers (Researcher 1, green; Researcher 2, blue) in the hippocampal subregions of the dorsal and ventral hippocampus. (A-F) Ventral hippocampus: CA1 pyramidal layer (CA1P; A, B), CA3 pyramidal layer (CA3P; C, D), and granule cell layer (GCL; E, F); (G-L) Dorsal hippocampus: CA1P (G, H), CA3P (I, J), and GCL (K, L). For each subregion, the first plot (A, C, E, G, I, K) shows the mean primary cilia length per animal (*N* = 6), while the adjacent plot (B, D, F, H, J, L) displays all individual primary cilia measurements pooled from the same 6 mice. Statistical test was paired *t*-test. *N* = 6. Each data set represents measurements from ∼60 primary cilia per region for each animal for each researcher. Black bars represent the mean ± SD.

To assess the reproducibility of primary cilia length quantification using 3D reconstruction, two independent researchers measured primary cilia length in distinct hippocampal subregions of the dorsal and ventral hippocampus. Across all regions examined (CA1P, CA3P, and GCL), both researchers obtained highly comparable mean primary cilia lengths when averaged per animal as well as when all individual cilia were analyzed. No significant differences were detected between researchers in any subregion, indicating strong inter-observer reliability and consistency in cilia length measurements (Figure 7A-L).

## DISCUSSION

This study compared stereology-based 3D quantification and IMARIS 3D reconstruction for assessing the length of neuronal primary cilia within specific subregions of the hippocampus on the DAB-stained sections for primary cilia. Both the dorsal and ventral hippocampus were analyzed due to their distinct functional roles in cognition and emotion. While it is impossible to determine absolute biological accuracy because true in vivo cilia length cannot be recovered after tissue processing, stereology provides the most accurate and unbiased estimate within the processed tissue.

Our findings revealed no statistically significant differences in primary cilia length between the two methods across the CA1P, CA3P, and GCL subregions. Furthermore, Bland-Altman analysis confirmed a high degree of agreement between the two approaches, suggesting reliability in measuring cilia length. The study also demonstrated consistency in cilia length across individual animals and repeated measurements, indicating minimal intra-individual variability. These results validate the robustness of both techniques in assessing neuronal primary cilia length in the hippocampus on the DAB-stained sections for primary cilia.

Although differences in primary cilia length are frequently reported and often interpreted as biologically meaningful to brain function, it is important to note that the field still lacks direct evidence explaining why changes in length alter neuronal function.^31^^,^^32^ It remains a widely held hypothesis not an established mechanism that shorter or longer cilia necessarily modify signaling capacity. For example, the ∼3 µm cilia observed in granule cells may be fully functional despite being shorter than the ∼7 µm cilia in CA1 pyramidal neurons. Thus, while cilia length is a useful morphological parameter, the causal relationship between length and function remains unresolved. Because the functional impact of cilia length differences is still not well understood, we did not aim to draw mechanistic conclusions about the physiological relevance of shorter or longer cilia. Instead, the objective of our study was methodological, ie, to demonstrate that primary cilia length can be measured accurately on IHC-stained sections using either stereology-based quantification or 3D reconstruction, even when cilia are relatively short (eg, ∼3 μm). By establishing a reliable approach that does not rely on immunofluorescence labeling, we provide a practical tool for studies involving densely packed neuronal regions, in which visualization and segmentation of cilia can be technically challenging. Changes in hippocampal macro- and microstructures have been associated with neurodevelopmental/neuropsychiatric illnesses, ranging from autism spectrum disorder to depression and schizophrenia.^33^^,^^34^ However, standardized, high-precision methods for measuring cilia length in densely packed brain regions like the hippocampus are lacking. Due to the complexity and variation of cilia morphology across different tissues and especially across different brain regions, measurements of primary cilia length are technically challenging especially, in the hippocampus with high cellular density, which increases the risk of cilia overlap and complicates accurate measurement. Moreover, lower contrast and greater background in DAB-immunostained sections compared to immunofluorescence, makes visualization more difficult. Apart from that, tissue shrinkage during processing, potentially leading to underestimation of true cilia length if not properly corrected.

Traditional two-dimensional (2D) measurements, such as maximum projection images, are prone to user bias and often underestimate cilia length, especially in 3D tissue preparations.^25^ Measurements of the lengths of primary cilia in chondrocytes and kidney cells indicated that cilia are significantly shorter when using 2D maximum projection images compared to a 3D method.^25^ Our 3D quantification approach with NewCAST uses systematic random sampling and stereological principles, correcting for shrinkage by calibrating optical disector height based on full-thickness section analysis. The brain sections were sampled systematically and randomly across animals and included all three hippocampal subregions. A Z-plot (histogram) of the measured cilia lengths indicated that a thickness ranges from 2 μm to 12 μm (10-μm thickness) corresponded to full tissue penetration and accounted for linear shrinkage, resulting in a final thickness of 10 μm.^35^

The 3D reconstruction method with IMARIS employs high-resolution z-stacks and manual thresholding. A systematic series of z-stacks was captured on ACIII-stained sections, using the center of the section as the reference point (zero), with a 100× oil-immersion lens. The z-stack height ranged from 18 to 20 μm, effectively eliminating the impact of tissue shrinkage on the 3D reconstruction of the primary cilia thereby preserving its full longitudinal structure. Ultimately, both methods are user-friendly and equally effective in providing a high-precision and comprehensive measurement approach for determining primary cilia lengths. In all tested hippocampal subregions, there were no differences between the two methods, suggesting that both approaches for measuring primary cilia length are equally reliable and independent of subregion or level of the hippocampus (ventral/dorsal).

On the other hand, newly developed AI tools, which have the potential to efficiently analyze large quantities of data, are dependent on user expertise and consistent high-quality images. Recent automated image analysis tools, such as CiliaQ and detectCilia, offer high-throughput quantification for immunofluorescence datasets. While these tools are valuable for large-scale studies, they have limitations. Their performance depends on image quality and contrast and they are less effective on DAB-stained or low-contrast images. In densely packed tissues, automated segmentation may struggle with overlapping structures, leading to potential errors in estimations of cilia length. Both CiliaQ and detectCilia require high-quality, standardized input and may not be robust against variations in staining intensity or background noise.^36^^,^^37^

To address this challenge, we compared two 3D-based methods of measuring neuronal primary cilia length on IHC-stained sections. Unlike immunofluorescence (IF) images, which typically offer clearer visualization, IHC staining often presents challenges due to lower contrast and potential background staining. Additionally, tissue processing including dehydration steps in the IHC processing causes shrinkage of the tissue, which may affect the size of measured primary cilia.

Due to the intra-regional variations of hippocampal function and structure, we performed measurements in several hippocampal subregions. In both the CA1P and CA3P regions, dysfunction of primary cilia can disrupt the signaling pathways, leading to impaired synaptic plasticity, altered neuronal connectivity, and deficits in learning and memory.^38^ Furthermore, signaling through primary cilia has been found be to shape interneuronal connectivity during development. Therefore, disruption of cilia function could alter the balance between excitatory and inhibitory synapses, a mechanism that is believed to be underlie neurodevelopmental disorders, such as autism spectrum disorder and schizophrenia.^31^ While the GCL has been associated with neurogenesis, it may play an important role in the underlying pathology of several psychiatric and neurodevelopmental disorders.^39^ Moreover, the dorsal and ventral parts of the hippocampus have different functions; while the dorsal part has been associated with cognitive functions, the ventral part seem to be involved in emotional behavior and stress response.^40–42^ Therefore, primary cilia length measurements need high precision to detect differences for tissue specific requirements. In the 3D reconstruction method, we did manual thresholding of the 3D image object but found no intra-variability when tested by three measurements with the same observer.

The analysis of primary cilia in mouse neocortical neurons, using fluorescence labeling for ACIII and electron microscopy, revealed that the lower cellular density of the neocortex resulted a less crowded environment, allowing for more reliable and accurate measurement of primary cilia length.^43^ A previous study highlighted an important challenge, ie, the potential overlap of primary cilia that come into contact making them appear as a single elongated structure in 2D maximum projection images.^25^ In contrast, our study focused on the densely packed pyramidal and granular cell layers of the hippocampus, where high cellular density complicates precise cilia measurement due to structural overlap and reduced visual clarity in IHC and IF stained sections. However, using 3D reconstruction and quantification methods, we were able to clearly distinguish the cilia as separate structures, ensuring accurate visualization and measurement.

## CONCLUSION

Using a dual 3D-method approach, we showed that 3D reconstruction produces cilia length measurements highly consistent with stereology-based quantification. Stereology remains the methodological gold standard due to its unbiased sampling and correction for tissue shrinkage while 3D image reconstruction offers a practical and precise complementary method. Both techniques are well-suited for primary cilia length measurements in densely packed neuronal layers. The two methods showed high agreement, with no systematic bias and narrow limits of error. Repeatability and reproducibility tests confirmed consistent cilia measurements across three trials, indicating minimal intra-individual variability.

## Supplementary Material

nlag001_Supplementary_Data

## Data Availability

The datasets analyzed during the current study available from the corresponding author on reasonable request.

## References

[1] Goetz SC , Anderson KV. The primary cilium: a signalling centre during vertebrate development. Nat Rev Genet. 2010;11:331-344. 20395968 10.1038/nrg2774 PMC3121168

[2] Nachury MV , Mick DU. Establishing and regulating the composition of cilia for signal transduction. Nat Rev Mol Cell Biol. 2019;20:389-405. 30948801 10.1038/s41580-019-0116-4 PMC6738346

[3] Walz G. Role of primary cilia in non-dividing and post-mitotic cells. Cell Tissue Res. 2017;369:11-25. 28361305 10.1007/s00441-017-2599-7 PMC5487853

[4] Kirschen GW , Xiong Q. Primary cilia as a novel horizon between neuron and environment. Neural Regen Res. 2017;12:1225-1230. 28966631 10.4103/1673-5374.213535 PMC5607811

[5] Waters AM , Beales PL. Ciliopathies: an expanding disease spectrum. Pediatr Nephrol. 2011;26:1039-1056. 21210154 10.1007/s00467-010-1731-7 PMC3098370

[6] Koemeter-Cox AI , Sherwood TW, Green JA, et al Primary cilia enhance kisspeptin receptor signaling on gonadotropin-releasing hormone neurons. Proc Natl Acad Sci USA. 2014;111:10335-10340. 24982149 10.1073/pnas.1403286111 PMC4104922

[7] Rhee S , Kirschen GW, Gu Y, et al Depletion of primary cilia from mature dentate granule cells impairs hippocampus-dependent contextual memory. Sci Rep. 2016;6:34370. 27678193 10.1038/srep34370 PMC5039642

[8] Wang W , Jack BM, Wang HH, et al Intraflagellar transport proteins as regulators of primary cilia length. Front Cell Dev Biol. 2021;9:661350. 34095126 10.3389/fcell.2021.661350 PMC8170031

[9] Kobayashi Y , Tomoshige S, Imakado K, et al Ciliary GPCR-based transcriptome as a key regulator of cilia length control. FASEB Bioadv. 2021;3:744-767. 34485842 10.1096/fba.2021-00029 PMC8409570

[10] Macarelli V , Leventea E, Merkle FT. Regulation of the length of neuronal primary cilia and its potential effects on signalling. Trends Cell Biol. 2023;33:979-990. 37302961 10.1016/j.tcb.2023.05.005 PMC7615206

[11] Baek H , Shin HJ, Kim JJ, et al Primary cilia modulate TLR4-mediated inflammatory responses in hippocampal neurons. J Neuroinflammation. 2017;14:189. 28927423 10.1186/s12974-017-0958-7 PMC5606072

[12] Oh EC , Vasanth S, Katsanis N. Metabolic regulation and energy homeostasis through the primary cilium. Cell Metab. 2015;21:21-31. 25543293 10.1016/j.cmet.2014.11.019 PMC4370781

[13] Ávalos Y , Hernández-Cáceres MP, Lagos P, et al Palmitic acid control of ciliogenesis modulates insulin signaling in hypothalamic neurons through an autophagy-dependent mechanism. Cell Death Dis. 2022;13:659. 35902579 10.1038/s41419-022-05109-9 PMC9334645

[14] Kozminski KG , Johnson KA, Forscher P, et al A motility in the eukaryotic flagellum unrelated to flagellar beating. Proc Natl Acad Sci USA. 1993;90:5519-5523. 8516294 10.1073/pnas.90.12.5519 PMC46752

[15] Marshall WF , Rosenbaum JL. Intraflagellar transport balances continuous turnover of outer doublet microtubules: implications for flagellar length control. J Cell Biol. 2001;155:405-414. 11684707 10.1083/jcb.200106141 PMC2150833

[16] Nachury MV , Seeley ES, Jin H. Trafficking to the ciliary membrane: how to get across the periciliary diffusion barrier? Annu Rev Cell Dev Biol. 2010;26:59-87. 19575670 10.1146/annurev.cellbio.042308.113337 PMC2952038

[17] Plotnikova OV , Pugacheva EN, Golemis EA. Aurora A kinase activity influences calcium signaling in kidney cells. J Cell Biol. 2011;193:1021-1032. 21670214 10.1083/jcb.201012061 PMC3115793

[18] Shin J , Tighanimine K, Cormerais Y, et al Growth factor-independent mTORC1 signaling promotes primary cilia length via suppression of autophagy. iScience. 2025;28:114204. 41476940 10.1016/j.isci.2025.114204 PMC12752765

[19] Badano JL , Mitsuma N, Beales PL, et al The ciliopathies: an emerging class of human genetic disorders. Annu Rev Genomics Hum Genet. 2006;7:125-148. 16722803 10.1146/annurev.genom.7.080505.115610

[20] Halbritter J , Bizet AA, Schmidts M, et al; UK10K Consortium. Defects in the IFT-B component IFT172 cause Jeune and Mainzer-Saldino syndromes in humans. Am J Hum Genet. 2013;93:915-925. 24140113 10.1016/j.ajhg.2013.09.012 PMC3824130

[21] Sorokin S. Centrioles and the formation of rudimentary cilia by fibroblasts and smooth muscle cells. J Cell Biol. 1962;15:363-377. 13978319 10.1083/jcb.15.2.363 PMC2106144

[22] Papon JF , Coste A, Roudot-Thoraval F, et al A 20-year experience of electron microscopy in the diagnosis of primary ciliary dyskinesia. Eur Respir J. 2010;35:1057-1063. 19840971 10.1183/09031936.00046209

[23] Bishop GA , Berbari NF, Lewis J, et al Type III adenylyl cyclase localizes to primary cilia throughout the adult mouse brain. J Comp Neurol. 2007;505:562-571. 17924533 10.1002/cne.21510

[24] Bansal R , Engle SE, Kamba TK, et al Artificial intelligence approaches to assessing primary cilia. J Vis Exp. 2021;(171):10.3791/62521. 10.3791/62521 PMC8791558 33999029

[25] Saggese T , Young AA, Huang C, et al Development of a method for the measurement of primary cilia length in 3D. Cilia. 2012;1:11. 23351171 10.1186/2046-2530-1-11 PMC3555708

[26] Brewer KM , Engle SE, Bansal R, et al Physiological condition-dependent changes in ciliary GPCR localization in the brain. eNeuro. 2023;10:ENEURO.0360-22.2023. 10.1523/ENEURO.0360-22.2023 PMC10012409 36849261

[27] Marcos R , Braganca B, Fontes-Sousa AP. Image analysis or stereology: which to choose for quantifying fibrosis? J Histochem Cytochem. 2015;63:734-736. 26033333 10.1369/0022155415592180 PMC4804732

[28] Kilkenny C , Browne WJ, Cuthill IC, et al Improving bioscience research reporting: the ARRIVE guidelines for reporting animal research. Osteoarthritis Cartilage. 2012;20:256-260. 22424462 10.1016/j.joca.2012.02.010

[29] Gundersen HJ. The smooth fractionator. J Microsc. 2002;207:191-210. 12230489 10.1046/j.1365-2818.2002.01054.x

[30] Ardalan M , Chumak T, Quist A, et al Reelin cells and sex-dependent synaptopathology in autism following postnatal immune activation. Br J Pharmacol. 2022;179:4400-4422. 35474185 10.1111/bph.15859 PMC9545289

[31] Guo J , Otis JM, Higginbotham H, et al Primary cilia signaling shapes the development of interneuronal connectivity. Dev Cell. 2017;42:286-300.e4. 28787594 10.1016/j.devcel.2017.07.010 PMC5571900

[32] Lee JE , Gleeson JG. Cilia in the nervous system: linking cilia function and neurodevelopmental disorders. Curr Opin Neurol. 2011;24:98-105. 21386674 10.1097/WCO.0b013e3283444d05 PMC3984876

[33] Marley A , von Zastrow M. A simple cell-based assay reveals that diverse neuropsychiatric risk genes converge on primary cilia. PLoS One. 2012;7:e46647. 23056384 10.1371/journal.pone.0046647 PMC3463515

[34] Chen X , Luo J, Leng Y, et al Ablation of Type III adenylyl cyclase in mice causes reduced neuronal activity, altered sleep pattern, and depression-like phenotypes. Biol Psychiatry. 2016;80:836-848. 26868444 10.1016/j.biopsych.2015.12.012 PMC5972377

[35] Mansouri M , Pouretemad H, Roghani M, et al Autistic-like behaviours and associated brain structural plasticity are modulated by oxytocin in maternally separated rats. Behav Brain Res. 2020;393:112756. 32535183 10.1016/j.bbr.2020.112756

[36] Hansen JN , Rassmann S, Stuven B, et al CiliaQ: a simple, open-source software for automated quantification of ciliary morphology and fluorescence in 2D, 3D, and 4D images. Eur Phys J E Soft Matter. 2021;44:18. 33683488 10.1140/epje/s10189-021-00031-y PMC7940315

[37] Budde-Sagert K , Krueger S, Sehlke C, et al detectCilia: an R package for automated detection and length measurement of primary cilia. Bioinform Biol Insights. 2024;18:11779322241280431. 39430098 10.1177/11779322241280431 PMC11490958

[38] Berbari NF , Bishop GA, Askwith CC, et al Hippocampal neurons possess primary cilia in culture. J Neurosci Res. 2007;85:1095-1100. 17304575 10.1002/jnr.21209

[39] Breunig JJ , Sarkisian MR, Arellano JI, et al Primary cilia regulate hippocampal neurogenesis by mediating sonic hedgehog signaling. Proc Natl Acad Sci USA. 2008;105:13127-13132. 18728187 10.1073/pnas.0804558105 PMC2529104

[40] Fanselow MS , Dong HW. Are the dorsal and ventral hippocampus functionally distinct structures? Neuron. 2010;65:7-19. 20152109 10.1016/j.neuron.2009.11.031 PMC2822727

[41] Moser MB , Moser EI, Forrest E, et al Spatial learning with a minislab in the dorsal hippocampus. Proc Natl Acad Sci USA. 1995;92:9697-9701. 7568200 10.1073/pnas.92.21.9697 PMC40869

[42] Henke PG. Hippocampal pathway to the amygdala and stress ulcer development. Brain Res Bull. 1990;25:691-695. 2289157 10.1016/0361-9230(90)90044-z

[43] Arellano JI , Guadiana SM, Breunig JJ, et al Development and distribution of neuronal cilia in mouse neocortex. J Comp Neurol. 2012;520:848-873. 22020803 10.1002/cne.22793 PMC3325766

