# Supplementary material for: Three-dimensional approaches to measuring primary cilia in hippocampal neurons: A comparative analysis
Source: J Neuropathol Exp Neurol. 2026 Jan 27;85(7):756–67. doi: 10.1093/jnen/nlag001 (PMC13293241; doi:10.1093/jnen/nlag001)
Supplement: nlag001_Supplementary_Data [file nlag001_supplementary_data.docx]

**Supplementary Table 1. Primary cilia length across hippocampal subregions measured by stereology-based 3D quantification and 3D reconstruction**

| Hippocampal Subregion | Method | Mean (µm) | SD | Min (µm) | Max (µm) | N |
| --- | --- | --- | --- | --- | --- | --- |
| CA1P – Dorsal | 3D-quantification | 7.47 | 0.27 | 7.12 | 7.83 | 6 |
|  | 3D-reconstruction | 7.66 | 0.38 | 7.00 | 8.11 | 6 |
| CA1P – Ventral | 3D-quantification | 7.73 | 0.28 | 7.34 | 8.14 | 6 |
|  | 3D-reconstruction | 7.62 | 0.71 | 7.11 | 9.00 | 6 |
| CA3P – Dorsal | 3D-quantification | 6.36 | 0.78 | 5.06 | 7.23 | 6 |
|  | 3D-reconstruction | 5.99 | 0.56 | 5.22 | 6.72 | 6 |
| CA3P – Ventral | 3D-quantification | 6.54 | 0.42 | 5.78 | 6.95 | 6 |
|  | 3D-reconstruction | 5.93 | 0.51 | 5.32 | 6.59 | 6 |
| GCL – Dorsal | 3D-quantification | 3.25 | 0.19 | 2.99 | 3.47 | 6 |
|  | 3D-reconstruction | 3.19 | 0.15 | 3.04 | 3.40 | 6 |
| GCL – Ventral | 3D-quantification | 3.61 | 0.49 | 2.75 | 4.18 | 6 |
|  | 3D-reconstruction | 3.35 | 0.34 | 2.92 | 3.97 | 6 |
